# Potential value and cardiovascular risks of programmed cell death in cancer treatment

**DOI:** 10.3389/fphar.2025.1615974

**Published:** 2025-07-03

**Authors:** Tian Yue, Dezhi Zheng, Jiali Yang, Jian He, Jun Hou

**Affiliations:** ^1^ Department of Cardiology, The Third People’s Hospital of Chengdu, Cardiovascular Disease Research Institute of Chengdu, The Affiliated Hospital of Southwest Jiaotong University, Chengdu, China; ^2^ Department of Cardiovascular Surgery, The 960th Hospital of the PLA Joint Logistic Support Force, Jinan, Shandong, China; ^3^ School of Life Science and Engineering, Southwest Jiaotong University, Chengdu, Sichuan, China

**Keywords:** programmed cell death, necroptosis, pyroptosis, autophagy, ferroptosis, cardiovascular risk

## Abstract

Programmed cell death (PCD) is equally important for maintaining overall homeostasis as it is for cell proliferation. The dynamic balance between cell proliferation and PCD promotes the body’s continuous self-repair and self-renewal, thus achieving cellular homeostasis. However, when this balance is disrupted, such as through unrestricted cell proliferation or the inhibition of PCD, tumors may occur. Moreover, this inhibition of cell death is considered a major cause of tumor development and a key factor contributing to the poor efficacy of many tumor treatments. Nowadays, with the discovery of an increasing number of PCD modalities, such as necroptosis, pyroptosis, autophagy, ferroptosis, and cuproptosis, PCD has broken the traditional classification of “apoptotic necrosis.” It is also an evolutionary necessity to prevent systemic damage caused by blocking a single cell death pathway. A systematic study of PCD may provide new insights into the origin of malignant tumors, the sensitivity of normal and malignant cells to treatment, and the development of treatment resistance. However, treatment regimens that act on PCD all pose significant cardiovascular risks, including excessive apoptosis of cardiomyocytes, cardiac rhythm abnormalities, cardiac remodeling, and myocarditis, among others. Currently, research on cardiovascular risks in tumor treatment is still incomplete. In this review, we describe different types of cell death processes and their roles in tumorigenesis. At the same time, we also discuss the basic and clinical applications of PCD in tumor pathogenesis, prevention, and treatment, as well as the known or potential cardiovascular risks. This provides a theoretical basis for the continuous progress of PCD-based tumor treatments.

## Introduction

**FIGURE 1:**
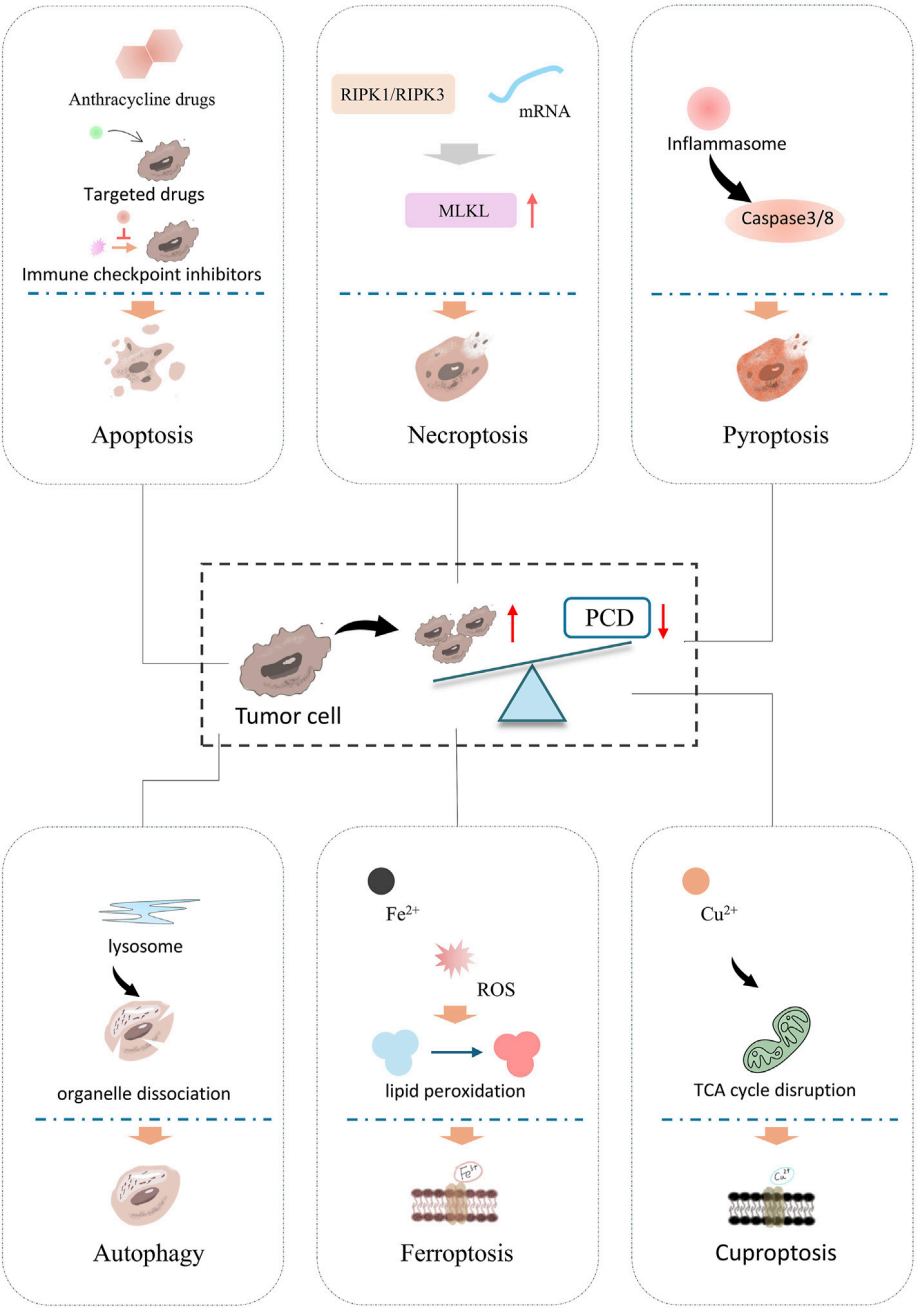
Classification of PCDs and main processes of different PCDs. PCD is a key target in tumor treatment mechanisms and drug development. It includes apoptosis, the classic form of programmed cell death, which depends on the caspase cascade reaction, resulting in cell shrinkage, nuclear fragmentation, and no inflammatory release. Necroptosis, key molecules, RIPK1 and RIPK3 (receptor-interacting protein kinases) phosphorylate MLKL (mixed lineage kinase-like protein). Pyroptosis is activated by the inflammasome, which activates Caspase-3/8, and the cleavage of Caspase-3/8 leads to cell rupture. Autophagy is mediated by lysosomes, followed by the encapsulation and degradation of organelles/protein. Ferroptosis, Fe^2+^ accumulation leads to ROS bursts and induces lipid peroxidation, causing cell membrane rupture. Cuproptosis, Cu^2+^ overload causes mitochondrial metabolic abnormalities (TCA cycle disruption), leading to protein lipidation aggregation and inducing cell death.

Inducing programmed cell death (PCD) appropriately represents a critical strategy in the development of anti-tumor drugs (Liu et al., 2022). Currently, cell death pathways are categorized into two major types: accidental cell death (ACD) and PCD (Radi, Stewart and O'Neil, 2018). ACD occurs following exposure to severe physical, chemical, or mechanical insults, with necrosis being its sole manifestation, observed in both infectious and noninfectious disease contexts (Peng et al., 2022). In contrast, PCD encompasses a diverse repertoire including apoptosis, necroptosis, pyroptosis, autophagy, ferroptosis, cuproptosis, and other modalities (Bertheloot et al., 2021; D'Arcy, 2019; Peng et al., 2022). As illustrated in Figure 1, PCD serves a pivotal role in maintaining homeostasis and regulating growth and development in multicellular organisms (Hsu et al., 2021; Wu et al., 2020). This process is fundamental to sustaining physiological balance, as both normal and tumor cells rely on PCD for self-renewal and growth regulation (Gao et al., 2022). Importantly, research has linked impaired PCD to tumorigenesis, disease progression, and the development of drug resistance across multiple cancer. Consequently, the targeted induction and activation of PCD have emerged as a focal area in oncotherapy (Tong et al., 2022).

Notably, many anti-tumor agents including classical alkylating agents, targeted therapies, and immune checkpoint inhibitors-exert their effects through modulation of PCD pathways (Kciuk et al., 2024; Wang et al., 2024b; Wu et al., 2023; Xiao et al., 2021a). However, clinical applications of chemotherapeutics, targeted drugs, and immune checkpoint inhibitors have revealed significant cardiovascular risks (Cai et al., 2022; Hashimoto et al., 2022; Wang et al., 2024a). Alarmingly, these treatment-related cardiovascular complications lead cancer patients to die of cardiovascular disease rather than the primary malignancy. (Sayour et al., 2024). This highlights the necessity of incorporating cardiac function monitoring as a standard component of oncological care. This review systematically discusses the current understanding of PCD in tumor treatment, the clinical applications of related therapeutic agents, and their potential cardiovascular toxicities, aiming to provide a theoretical foundation for safeguarding cardiovascular health during cancer therapy.

## Basic studies and clinical studies

### Specific descriptions of PCD

Each of PCD is governed by distinct molecular regulatory networks and exhibits unique morphological and biochemical hallmarks (Yuan and Ofengeim, 2024), as summarized in Table 1. The evolution of multiple PCD represents an adaptive strategy for multicellular organisms. Throughout the lifecycle, innumerable “redundant” or “pathologically altered” cells must be eliminated (Kulkarni and Hardwick, 2023). Relying exclusively on a single death mechanism would pose critical risks: inhibition of such a pathway-whether due to genetic mutation, pathogen evasion, or therapeutic intervention-could disrupt cellular homeostasis, potentially leading to uncontrolled proliferation (as in cancer) or excessive inflammation. This functional redundancy in PCD therefore serves as a robust safeguard, ensuring that disruptions in one pathway can be compensated for by others, thereby maintaining organismal integrity across diverse physiological and pathological contexts (Tower, 2015).

**TABLE 1:** Summary of multiple cell death pathways.

Type of cell death	Key molecule	Morphological change
Necrosis	None	Cell swelling, membrane rupture, loss of organelle
Apoptosis	Bax, Bak, Caspase8, Caspase3, Caspase9	Cell shrinkage, collapse of subcellular structure, condensation of chromatin, fragmentation of nucleus, formation of plasma membrane vesicles
Necroptosis	TNFα, caspase-8, RIPK1, RIPK3, MLKL	Membrane rupture, expansion of cytoplasm and organelles, chromosome condensation, release of cellular contents
Pyroptosis	NLRs, ALRs, Caspase1, Caspase11	Cell swelling, membrane rupture, chromosome condensation and fragmentation
Autophagy	ULK1, ATGs, LC3, mTOR, Beclin1	Membrane integrity, autophagosomes and vesicles formed
Ferroptosis	Fe^2+^, GPX4, GSH, MDA,ROS,LPO	Diminutive mitochondria, decreased cristae, collapsed and ruptured membrane
Cuproptosis	Cu^2+^, FDX1,DLAT, LIAS	Membrane integrity, collapse of subcellular structure

Abbreviations in the table: Bax, BCL2-associated X; bak, BCL2 antagonist/killer; TNFα, Tumor Necrosis Factor α; RIPK1/3, Receptor Interacting Protein Kinases 1/3; MLKL, Mixed Lineage Kinase Domain-Like Protein; NLRs, Nucleotide-binding Leucine-rich Repeat Receptors; ALRs, AIM2-Like Receptors; ULK1, Unc-51, Like Autophagy Activating Kinase 1; ATGs, Autophagy-Related Genes; LC3, Microtubule-Associated Protein 1 Light Chain 3; mTOR, mechanistic target of rapamycin; GPX4, Glutathione Peroxidase 4; GSH, glutathione; MDA, malondialdehyde; ROS, reactive oxygen species; LPO, lipid peroxidation; FDX1, Ferredoxin 1; DLAT, dihydrolipoamide acetyltransferase; LIAS, lipoyl synthase.

### Apoptosis

**FIGURE 2:**
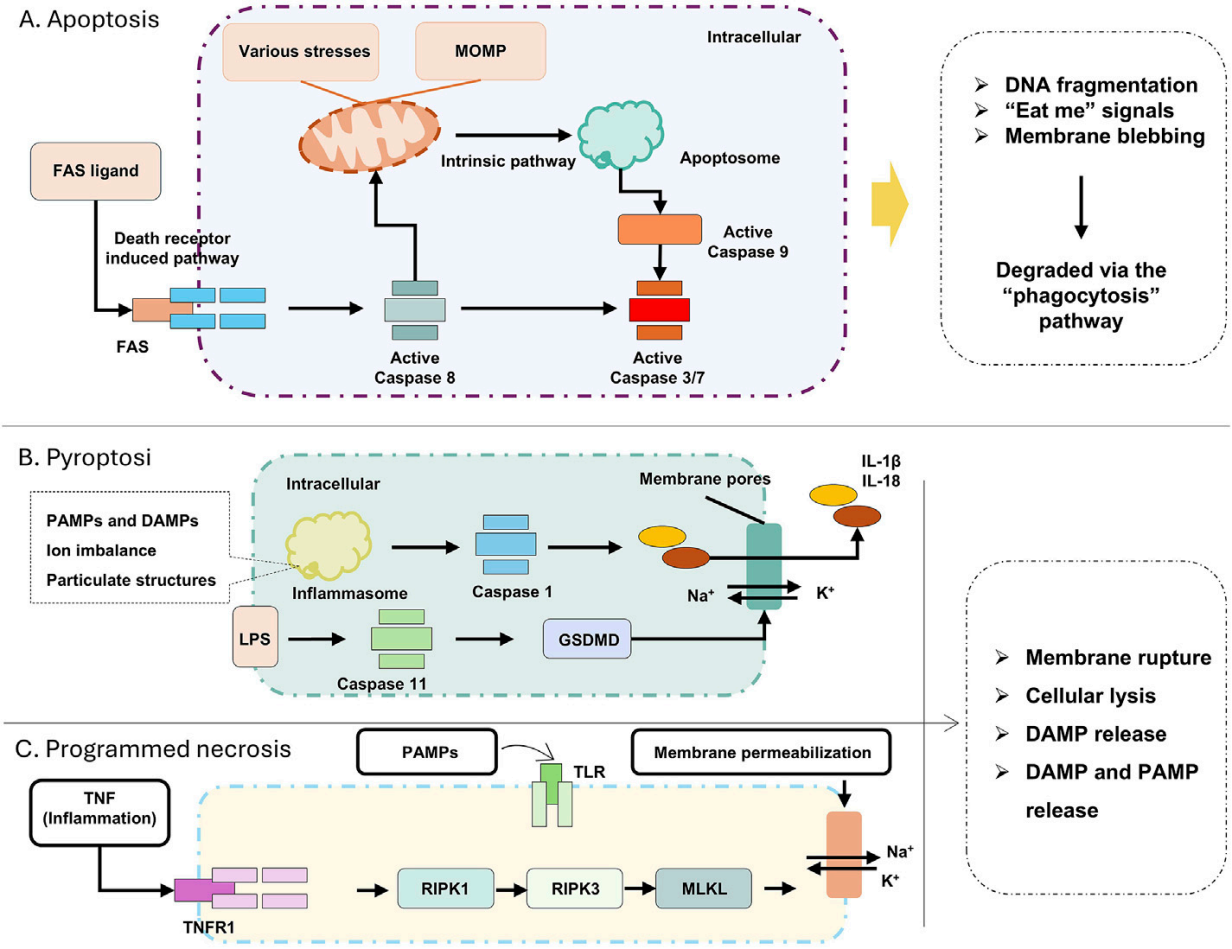
**(A)** Apoptosis related pathways; **(B)** Pyroptosis startup and process; **(C)** Related pathways of Programmed necrosis. Apoptosis can be triggered by either extrinsic or intrinsic pathways. The death receptor pathway is activated by ligands on the plasma membrane (e.g., FAS ligand), leading to the activation of caspase-8. The intrinsic pathway can be induced by a wide range of stress stimuli, including DNA damage or withdrawal of growth factors, as well as developmental cues. This cell death pathway is associated with mitochondrial outer membrane permeabilization (MOMP), the release of cytochrome c, and the activation of pro-caspase 9. Both apoptotic pathways converge on the proteolytic activation of effector caspases 3 and 7 by pro-caspases 8 and 9. Necrotic apoptosis and pyroptosis are the primary pro-inflammatory lytic forms of PCD. They are associated with cell lysis, which, in the context of pathogen infection, leads to the release of damage-associated molecular patterns (DAMPs) and pathogen-associated molecular patterns (PAMPs). DAMPs and PAMPs are recognized by neighboring phagocytes, inducing necrotic apoptosis (and certain other signals) through stimulation of TNF receptor 1 (TNFR1) or Toll-like receptors (TLRs), leading to the activation of receptor-interacting serine/threonine kinases RIPK 1 and RIPK 3. This causes conformational changes and the activation of the mixed lineage kinase-like (MLKL) pseudo kinase, which then translocate to the cell membrane, where it induces membrane rupture.

Apoptosis is a caspase-dependent form of PCD that occurs under defined physiological or pathological conditions (Kesavardhana et al., 2020; Obeng, 2021). Apoptosis proceeds without cell membrane rupture, a critical difference from necrosis that minimizes collateral tissue damage (D'Arcy, 2019). Apoptosis is generally classified into two categories: the extrinsic apoptosis pathway and the intrinsic apoptosis pathway. The extrinsic apoptosis pathway, referred to in the text as the death receptor pathway, is triggered by the binding of extracellular death ligands to death receptors on the cell surface, which in turn activates a series of caspase proteases, leading to cell apoptosis (Carneiro and El-Deiry, 2020; D'Arcy, 2019; Hu et al., 2018). B-cell lymphoma/leukemia-2 protein (BCL-2) protein is a core member of the BCL-2 family and plays a key regulatory role in apoptosis, especially in the intrinsic apoptosis pathway (Kaufman et al., 2021). The intrinsic apoptosis pathway is activated when cells are exposed to internal stress factors (such as DNA damage, oxidative stress, protein misfolding, etc.). This leads to changes in mitochondrial function and structure, alterations in mitochondrial membrane permeability, and the release of substances such as cytochrome c into the cytoplasm. This, in turn, activates caspase proteases, triggering apoptosis (Figure 2A) (Kesavardhana et al., 2020; Warren et al., 2019; Zhang et al., 2019).

When the heart is stimulated by chemotherapeutic drugs it results in the release of large amounts of reactive oxygen species (ROS) and activates oxidative stress in cardiomyocytes. Excessive oxidative stress damage the DNA, proteins and lipids of cardiomyocytes and activates the apoptotic signaling pathway of cardiomyocytes, leading to apoptosis of cardiomyocyte (Hof et al., 2019; Peng et al., 2024). Cardiomyocytes are the main functional units of cardiac contraction and diastole, and massive apoptosis of cardiomyocytes leads to a decrease in cardiac contractility and a series of adverse effects, such as arrhythmia, ventricular remodeling, and ultimately, heart failure (HF) (Pan et al., 2024).

### Pyroptosis

Pyroptosis is a pro-inflammatory form of PCD executed via canonical or noncanonical pathways, distinguished by the formation of plasma membrane pores and release of pro-inflammatory cytokines (Shi et al., 2017; Yu et al., 2021). The canonical pathway is initiated by pathogen-associated molecular patterns (PAMPs) or Damage-Associated Molecular Patterns (DAMPs) activating cytosolic pattern recognition receptors (PRRs), such as nucleotide-binding oligomerization domain-like receptors (NLRs). Upon ligand recognition, NLRs oligomerize with apoptosis-associated speck-like protein containing a caspase-recruitment domain (ASC) to form inflammasomes, which recruit and activate pro-caspase-1 (Fang et al., 2020; Shi et al., 2015). Activated caspase-1 then cleaves the gasdermin D (GSDMD) protein, releasing its N-terminal domain that translocates to the plasma membrane, forming pores that induce osmotic lysis (Zhou et al., 2020). Concurrently, caspase-1 processes pro-interleukin-1β (pro-IL-1β) and pro-IL-18 into their active forms, which are released through the pores to propagate inflammatory responses (Sarhan et al., 2018). The noncanonical pathway involves cytosolic detection of bacterial lipopolysaccharides (LPS) by caspase-4/5 (in humans) or caspase-11, leading to GSDMD activation and subsequent pore formation without inflammasome assembly, though it also results in IL-1β/IL-18 release and cell membrane rupture (Christgen et al., 2020). This tightly regulated process links microbial infection or cellular stress to controlled inflammation, highlighting pyroptosis as a critical interface between innate immunity and RCD (Figure 2B).

Beyond GSDMD, the Gasdermin family member GSDME has been recognized as an alternative executor of pyroptosis. Under physiological or pathological conditions, GSDME can be cleaved by caspase-3, caspase-8, or granzyme A, liberating its N-terminal domain to form membrane pores and induce pyroptotic cell death (Sarhan et al., 2018; Zhou et al., 2020). Consequently, a defining characteristic of pyroptosis is the proteolytic activation of Gasdermin proteins, whose N-terminal fragments oligomerize to form membrane-disrupting pores, ultimately leading to lytic cell death and the release of inflammatory mediators.

### Programmed necrosis

Programmed cell death, necroptosis, when it used as a technical term, represents a caspase-independent modality of PCD that is activated when canonical apoptotic pathways are functionally inhibited (Gong et al., 2019). Morphologically, this process is characterized by features of necrosis, including loss of plasma membrane integrity, cytoplasmic and organellar swelling, chromatin condensation, and release of intracellular components, such as DAMPs, proinflammatory cytokines, and chemokines-thereby instigating a robust inflammatory response *in vivo* (Figure 2C) (Bertheloot et al., 2021; Gong et al., 2019; Snyder and Oberst, 2021).

Necroptosis is executed through a tightly regulated signaling cascade involving receptor-interacting protein kinases 1 and 3 (RIPK1, RIPK3) and mixed-lineage kinase domain-like protein (MLKL). Activation initiates when RIPK1 and RIPK3 assemble via their RIP homotypic interaction motifs (RHIMs), undergoing sequential autophosphorylation and trans-phosphorylation to form a functional necrosome complex. Phosphorylated RIPK3 then recruits and phosphorylates MLKL, triggering its oligomerization and translocation to the plasma membrane and intracellular organelle membranes. At these sites, phosphorylated MLKL forms pore-forming complexes in biological membranes, leading to membrane rupture, cytoplasmic content leakage into the extracellular space, and the characteristic inflammatory phenotype of necroptosis. Concurrently, this process releases DAMPs, DAMPs are endogenous molecular patterns released by damaged or dying cells during injury, activating immune cells like macrophages and triggering inflammatory responses, including interleukin-1α (IL-1α), IL-1β, and IL-33, which act as danger signals to recruit immune cells to sites of tissue injury, facilitating clearance of necroptotic cells and orchestrating local inflammatory responses (Martens et al., 2021; Zhao et al., 2012).

When Programmed Necrosis is used as a broad concept, it refers to a genetically regulated process of cell death characterized by features like necrosis. It encompasses various specific forms of cell death, including necroptosis, pyroptosis, and ferroptosis, among others, which will not be elaborated upon here. Mitochondrial-dependent necrosis is a specific form of necrosis that is typically closely associated with the opening of the mitochondrial permeability transition pore (mPTP). When the mPTP opens, it leads to mitochondrial dysfunction, such as the cessation of ATP synthesis driven by respiration and the collapse of the mitochondrial membrane potential, ultimately triggering cell necrosis. This represents an important pathway within Programmed Necrosis. (Chen et al., 2023).

### Autophagy

**FIGURE 3:**
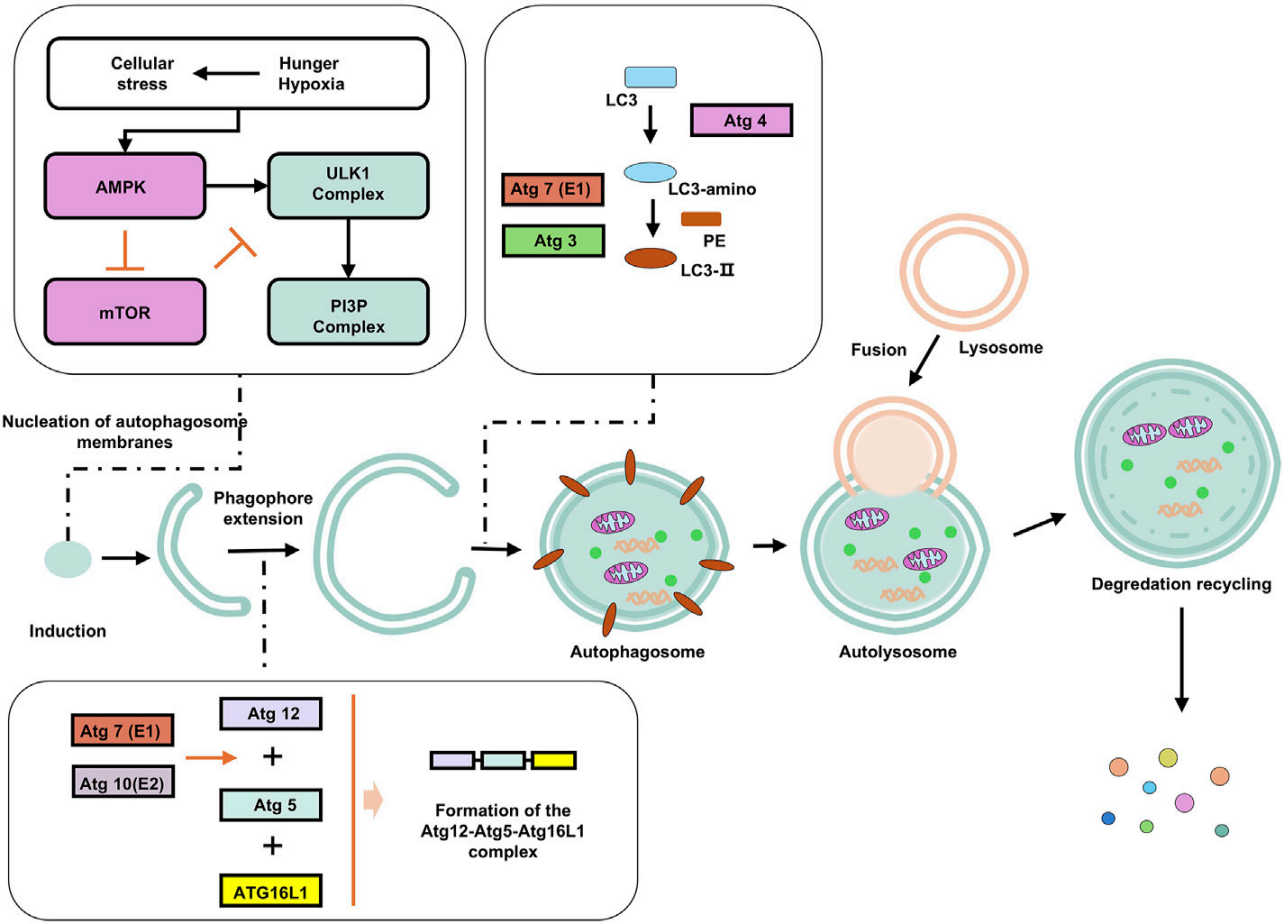
Autophagy and its influencing factors. Under starvation, hypoxia, and other stress conditions, AMPK is phosphorylated and inhibits mTORC1 (mammalian target of rapamycin complex 1), relieving its inhibition on autophagy. The activated ULK1 phosphorylates Beclin-1 (a key autophagy protein), enabling it to bind to VPS34 (class III PI3K) to form a PI3K complex. This complex catalyzes phosphatidylinositol (PI) to generate phosphatidylinositol-3-phosphate (PI3P), marking the formation site of the autophagosome. Atg12 is catalyzed by E1 (Atg7) and E2 (Atg10) enzymes, binds to Atg5, and then forms a multimer with Atg16L1, which localizes to the autophagosome membrane and promotes membrane elongation. LC3 (LC3-I) in the cytoplasm is cleaved by Atg4 to expose the C-terminal glycine, and then catalyzed by Atg7 (E1) and Atg3 (E2) to bind to phosphatidylethanolamine (PE) to form LC3-II, which anchors to the autophagosome membrane and promotes membrane closure. Subsequently, the autophagosome fuses with the lysosome. Enzymes in the autolysosome decompose macromolecules into small molecules such as amino acids and fatty acids, which are released into the cytoplasm through transport proteins on the lysosomal membrane for reuse by the cell.

Autophagy enables cells to adapt to stress or injury by degrading cytoplasmic constituents (Dikic and Elazar, 2018; Glick et al., 2010). Morphologically and mechanistically, autophagy is defined by the enzymatic degradation of proteins and organelles via lysosomal hydrolases, and the formation of double-membrane vesicles reflecting dynamic rearrangement of cellular membranes (Figure 3) (Glick et al., 2010).

Autophagy patterns represent a range of conserved strategies for cellular quality control, nutrient cycling, and stress adaptation. While macroautophagy is usually associated with non-selective bulk degradation (although selective degradation via cargo receptors such as p62/SQSTM1 is possible), microautophagy and CMA show a higher degree of specificity, highlighting the functional diversity of autophagic processes in maintaining cellular homeostasis and coordinating PCD.

### Ferroptosis

**FIGURE 4:**
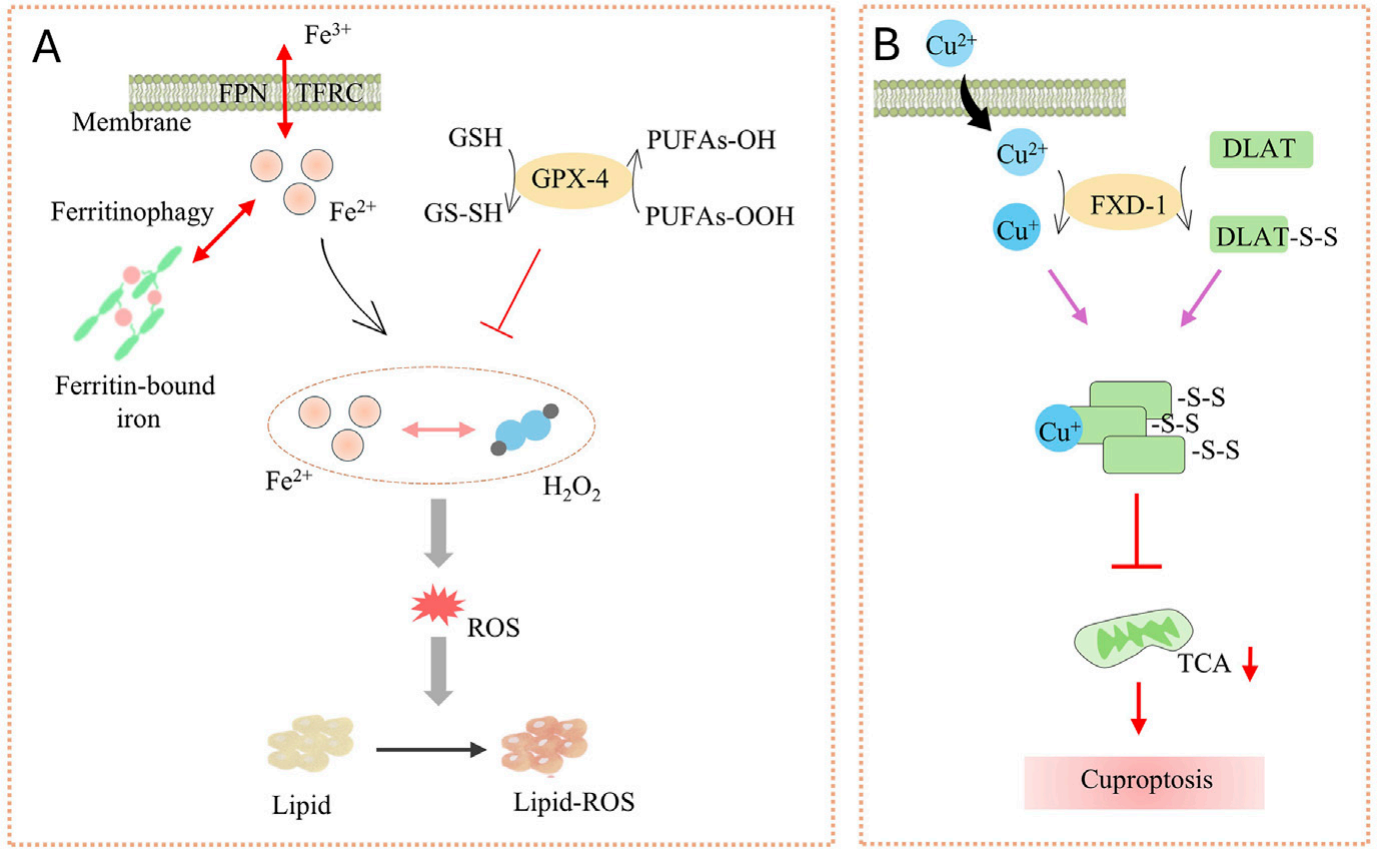
**(A)** The launch and key factors of Ferroptosis; **(B)** Cuproptosis process. Ferroportin (FPN) and TFRC (Coding for transferrin receptor 1) on the membrane regulate Fe^2+^, while ferritinophagy promotes the release of Fe^2+^ from ferritin-bound iron. Fe^2+^ reacts with H_2_O_2_ to generate ROS. GSH and GPX-4 can inhibit the production of PUFAs-OOH. ROS triggers lipid peroxidation (Lipid-ROS), inducing ferroptosis. Cu^2+^ enters the cell through the membrane and interacts with FXD-1 to induce the formation of disulfide bonds (DLAT-S-S) in DLAT, disrupting the TCA cycle and ultimately leading to cuproptosis.

Dr. Brent R Stockwell first introduced the concept of “iron metamorphosis” in 2012 (Dixon et al., 2012). This is a unique mode of cell death characterized by iron-dependent lipid peroxidation and massive accumulation of ROS. The central mechanism of the iron metabolic response lies in the induction of cell death by lipid peroxidation of unsaturated fatty acids highly expressed on cell membranes catalyzed by the action of divalent iron or ester oxygenases (Hirschhorn and Stockwell, 2019; Li and Li, 2020). In addition, the expression of antioxidant systems, namely glutathione (GSH) and glutathione peroxidase 4 (GPX4), is also inhibited (Figure 4A) (Li and Li, 2020).

In the cell membrane, phospholipids produce lipid ROS through non-enzymatic (Fenton reaction) or enzymatic (lipid oxidase) processes, while iron acts as a catalyst. These lipid ROS accumulate and undergo peroxidation with polyunsaturated fatty acids (PUFA) in the cell membrane. This ultimately leads to cell membrane rupture and cell death (Jiang et al., 2021; Li and Li, 2020).

### Cuproptosis

Cuproptosis, the central mechanism is that excessive intracellular accumulation of copper (Cu^2+^)-through copper ion imports or dysregulated mitochondrial respiration-binds to the key enzyme in the pyruvate dehydrogenase complex, thioacylated dihydrolipoamide S-acetyltransferase (DLAT). This interaction induces DLAT oligomerization and aggregation, leading to the formation of insoluble protein clusters that disrupt mitochondrial metabolism and trigger cytotoxicity, ultimately leading to cell death (Figure 4B) (Tsvetkov et al., 2022).

## Treatment regimens/targets and cardiovascular risks for different types of PCD

### Cardiovascular risks for various PCD-targeting cancer treatment regimens

Apoptosis has long been recognized as an important mechanism to prevent tumorigenesis, and inhibition of apoptosis is one of the characteristics of tumor cells (Hanahan and Weinberg, 2011). The molecular mechanisms that inhibit apoptosis in tumor cells include disrupting the balance between pro- and anti-apoptotic proteins (Bertrand et al., 2008; Goolsby et al., 2005; Mohamad Anuar et al., 2020), inhibiting caspase activity (Jung et al., 2021; Sangaran et al., 2021), and disrupting death receptor signaling (Conlon et al., 2020; Xu et al., 2021). Caspases have been found to be key proteins in the initiation and execution of apoptosis, and their reduced expression or impaired function is closely associated with tumor progression (Kesavardhana et al., 2020). Loss of caspase 3 expression and function has been found to promote the survival of a variety of tumor cells (Devarajan et al., 2002).

#### Chemotherapy

Chemotherapy is the longest and most widely used drugs in tumor treatment, such as cyclophosphamide, fluorouracil, adriamycin (DOX), and some plant bases. Chemotherapy is usually aimed at interfering with the growth and division of tumor cells, including interfering with the DNA synthesis and protein synthesis processes of tumor cells to achieve tumor suppression (Peng et al., 2024). Since chemotherapeutic agents act on rapidly growing and dividing cells, they inevitably harm cells that are metabolically active, causing side effects such as myelosuppression, alopecia, liver and kidney damage, and cardiomyocyte damage (Hof et al., 2019). The process of chemotherapy is closely related to PCD. Currently, radiotherapy and chemotherapy attempt to induce apoptosis by activating the killing potential of caspase 3. However, new studies have shown that caspase 3 is not an oncogenic but a pro-oncogenic factor after cells are exposed to chemicals and radiation (Yosefzon et al., 2018). It has been shown that caspase 3 is involved in promoting tumor repopulation after radiotherapy through a paracrine signaling pathway (Cheng et al., 2019). In addition, caspase 3 promotes tumor growth by providing a pro-angiogenic microenvironment. Researchers have found that colon tumor patients with low caspase 3 activation have longer disease-free survival (Kunac et al., 2019).

#### Targeting drug

Human Epidermal Growth Factor Receptor 2 (HER2) inhibitors, as classical tumor-targeting agents, are not described in detail here. The Bcl-2 family plays key regulatory roles by inhibiting apoptosis and promoting apoptosis. Bcl-2 and/or Bcl-XL are overexpressed in many human tumors such as gastrointestinal tumors, lymphomas, neuroblastomas, and bladder cancers. High expression of Bcl-2 inhibits apoptosis and accelerates cell growth, which in turn leads to malignancy (Song et al., 2021; Tao et al., 2021). The anti-apoptotic protein Bcl-2 has become an emerging drug target in cancer therapy (Hanahan and Weinberg, 2011; Song et al., 2021). In the past 20 years, various inhibitors have been introduced, and some drugs have entered the clinical stage. Statistically, some Bcl-2 inhibitors have entered the clinical study stage, which are Oblimersen, Navitoclax (ABT-263), Venetoclax (ABT-199), and Obataclax mesylate (GX15-070), among which Venetoclax has been approved for marketing in 2016 (DiNardo et al., 2020; Jiménez-Guerrero et al., 2018; Pullarkat et al., 2021; Walker et al., 2021; Wei et al., 2020). Impaired death receptor signaling pathway is also one of the mechanisms by which tumor cells evade apoptosis. Studies have shown that tumor cells acquire drug resistance by downregulating the expression of death receptors. For example, reduced expression of CD95 inhibits the apoptotic pathway, leading to resistance to chemotherapy in patients with leukemia or neuroblastoma (Friesen et al., 1997; Fulda et al., 1998).

#### Immune checkpoint inhibitors (ICIs)

ICIs potentiate systemic immune responses, particularly T cell-mediated antitumor immunity, by abrogating tumor-induced immunosuppression and can also induce tumor cell apoptosis via extrinsic death receptor pathways (Carlino et al., 2021). Since the 2011 FDA approval of the CTLA-4 inhibitor ipilimumab, the first ICIs for melanoma numerous ICIs have entered clinical trials or achieved market launch, including the PD-1 inhibitor nivolumab and PD-L1 inhibitor atezolizumab. CTLA-4 is expressed on T cells and competes with the co-stimulatory molecule CD28 for binding to B7 molecules on antigen-presenting cells. This interaction inhibits T-cell activation. CTLA-4 inhibitors like ipilimumab block CTLA-4, enhancing T-cell activation and promoting an immune response against tumor cells. PD-1 also acts on T cells, PD-1 is expressed on the surface of activated T cells, PD-L1 is often overexpressed on tumor cells. When PD-1 binds to PD-L1, it sends an inhibitory signal to T cells, preventing them from attacking tumor cells. PD-1 inhibitors, such as pembrolizumab and nivolumab, block the interaction between PD-1 and PD-L1, enabling T cells to regain their anti-tumor activity (Ghosh et al., 2021; Rittmeyer et al., 2017). Emerging ICIs in clinical development target additional pathways such as LAG-3, TIGIT, and TIM-3, expanding the therapeutic landscape of immune-oncology (Anderson et al., 2016).

### Necroptosis in tumor treatment

A dual role in both promoting and reducing tumor growth has been identified in various cancer (Qin et al., 2019). Necroptosis serves as a fail-safe mechanism of cell death in cells where apoptosis cannot be induced, thereby inhibiting tumor progression. However, necroptosis, as a form of PCD, can trigger inflammatory responses, which have been reported to promote cancer metastasis and immunosuppression (Najafov et al., 2017).

The RIPK1/RIPK3 complex, a critical regulator of necroptosis, has been found to be downregulated in samples from cancer patients, including colorectal cancer (Stoll et al., 2017), gastric cancer (Feng et al., 2015), acute myeloid leukemia (AML) (Nugues et al., 2014), and melanoma (Geserick et al., 2015). Additionally, RIPK1/RIPK3 has been implicated in colorectal cancer. In a cohort study involving over 100 patients, low RIPK3 expression was independently associated with reduced disease-free survival and overall survival (Feng et al., 2015). Similarly, low RIPK3 expression predicts poor prognosis in gastric cancer (DiNardo et al., 2020). These findings suggest that RIPK3 may exert anti-inflammatory and anti-tumor effects in cancer. However, in some animal models, RIPK1 inhibition has been linked to adverse cardiovascular effects, such as myocardial inflammation or structural cardiac alterations. Notably, excessive activation of the RIPK1 pathway may induce cardiac dysfunction (Beal et al., 2018). As the executioner of necroptosis, MLKL, upon activation, induces plasma membrane rupture and initiates the necroptotic process. Current research indicates that MLKL-encoded mRNA can promote MLKL expression in the tumor microenvironment, leading to necroptosis in tumor cells.

### Pyroptosis in cancer treatment

Extensive studies have demonstrated that pyroptosis is closely associated with the development and metastasis of various cancers. Prolonged exposure to an inflammatory environment increases the risk of cancer. Specifically, pyroptosis induces the release of cytokines such as IL-1 and IL-18, which can promote tumor invasion, thereby enhancing tumorigenesis and metastatic potential. Pyroptosis acts as a double-edged sword in cancer, capable of either promoting or suppressing tumorigenesis. While its pro-tumorigenic effects have been widely investigated, the relationship between pyroptosis and anti-cancer immunity remains incompletely understood (Yu et al., 2021).

In lung cancer, PPVI (a saponin derived from Trillium tschonoskii Maxim) inhibits the proliferation of NSCLC cells via the ROS/NF-κB/NLRP3/GSDMD signaling pathway, exhibiting significant anti-tumor effects. Given the low chemosensitivity of NSCLC, this mechanism suggests that PPVI may represent a novel therapeutic target for NSCLC in the future (Teng et al., 2020).

Currently, many chemotherapeutic agents exert anti-tumor effects by inducing pyroptosis. For instance, in triple-negative breast cancer, cisplatin (CDDP) appears to increase the complete pathological response rate and suppress tumor growth and metastasis *in vitro* and *in vivo* by activating the NLRP3/caspase-1/GSDMD-mediated pyroptosis pathway (Yan et al., 2021). Sorafenib, a kinase inhibitor, reduces MHC-I expression in HCC tumor cells. It has been reported to exert anti-tumor effects by inducing pyroptosis in macrophages. Beyond this indirect mechanism, pyroptosis also directly exhibits significant anti-tumor properties in HCC cells (Chen et al., 2022; Hage et al., 2019). In recent years, CAR-T therapy has demonstrated remarkable efficacy in treating tumors, particularly B-cell acute lymphoblastic leukemia (B-ALL), with complete remission rates reaching 90%. CAR-T cells can induce pyroptosis in primary B-ALL cells by rapidly releasing granzyme B, activating caspase-3 to cleave GSDME, and ultimately triggering pyroptosis (Zhang et al., 2021).

Recent studies have validated pyroptosis as a feasible anti-tumor immune mechanism with clinical potential. Many researchers are exploring combinations of pyroptosis with other cancer therapies to modulate pyroptosis and inhibit tumor cell proliferation, migration, and invasion. Potential targeted strategies include inhibiting NLRP3, caspase-1, caspase-3, and GSDMD (Li et al., 2021).

### Auxiliary role of autophagy in cancer treatment

Autophagy exerts a pronounced dual role in tumors, contributing to the limited development of autophagy-targeting antitumor drugs. Chloroquine (CQ) and hydroxychloroquine (HCQ) are among the few autophagy-targeting compounds that have entered clinical investigations (Levy et al., 2017). Their mechanism of action involves lysosomal deacidification, which prevents autophagosomes from fusing with lysosomes and achieving complete degradation of their cargo (Yang et al., 2013). The more detailed mechanism by which CQ induces lysosomal deacidification remains unclear. Preliminary clinical trials indicate that CQ or HCQ are well-tolerated, particularly without the neurodegenerative side effects observed in mice with knockout of certain autophagy-related (ATG) genes, which are associated with neurodegeneration (Ferreira et al., 2021). As a major metabolite of CQ, HCQ exhibits lower toxicity at peak concentrations, leading to its increased use in clinical trials. While overall efficacy reports are positive, most trials enroll a small number of patients, necessitating further validation of results (Alruwaili et al., 2023).

### Ferroptosis in cancer treatment

Researchers are increasingly recognizing that various conventional cancer therapies can induce ferroptosis, and enhancing ferroptosis induced by these treatments may further improve therapeutic efficacy. Radiotherapy (RT) triggers ferroptosis through multiple parallel mechanisms (Pearson et al., 2021). Following RT, tumor cells activate adaptive responses—such as upregulating SLC7A11 or GPX4 expression—to antagonize RT-induced ferroptosis. Thus, combining RT with FINs (ferroptosis inducers) targeting SLC7A11 or GPX4 increases sensitivity of tumor cells or xenograft tumors to radiation by enhancing ferroptotic susceptibility (Pearson et al., 2021). Similarly, the chemotherapeutic agent gemcitabine induces GPX4 expression and activity; GPX4 inhibition counteracts this effect, increasing gemcitabine sensitivity in tumor cells and xenografts via ferroptosis induction. The cardiovascular risks of gemcitabine are typically linked to factors such as dosage, treatment duration, and preexisting cardiovascular conditions, with reported symptoms including myocardial ischemia (e.g., chest pain, tightness), rarely progressing to myocardial infarction. Targeting SLC7A11 with FINs has also been shown to sensitize tumor cells to chemotherapy (e.g., CDDP, doxorubicin, DOX), immunotherapy (e.g., ICIs), and combined RT-immunotherapy (Lang et al., 2019).

Notably, in cancer with intrinsic or acquired treatment resistance, induction of ferroptosis by FINs restores sensitivity to conventional therapies. Mutations in tumor suppressors TP53 or KEAP1 evade RT-induced ferroptosis by upregulating SLC7A11 and other anti-ferroptotic mechanisms, leading to inherent radiation resistance (Zheng et al., 2022). Acquired radioresistant tumor cells also exhibit SLC7A11 upregulation and ferroptosis resistance; in these cells and xenografts, FINs reactivate ferroptotic sensitivity, restoring responsiveness to RT. Similarly, FINs can overcome chemoresistance in certain cancers: targeting SLC7A11 with FINs eliminates CDDP resistance in head and neck squamous cell carcinoma cells and xenografts (He et al., 2021), while *in vitro* studies show FIN-mediated SLC7A11 inhibition reverses docetaxel resistance in ovarian tumor cells (He et al., 2021).

### Cuproptosis in cancer treatment

Cuproptosis is a newly discovered copper-dependent form of PCD identified in 2022. Currently, research on drugs for treating tumors caused by copper death is still in its early stages. Elesclomol (ES) is a chemotherapeutic adjuvant developed by synta pharmaceuticals and originally developed for treating metastatic melanoma. Recent studies have found that its synergistic effect with copper ions can specifically induce cuproptosis by degrading iron-sulfur proteins and interfering with mitochondrial metabolism. ES is primarily used in cancer therapy research, with its mechanisms involving cuproptosis, oxidative stress-induced cell death, and mitochondrial function regulation. There are currently no reported explicit applications in the cardiovascular field. Cuproptosis is often combined with other PCD mechanisms (such as apoptosis and ferroptosis) or traditional therapies to overcome the limitations of single mechanisms.

### PCD-dependent oncology drugs and cardiovascular risks

**FIGURE 5:**
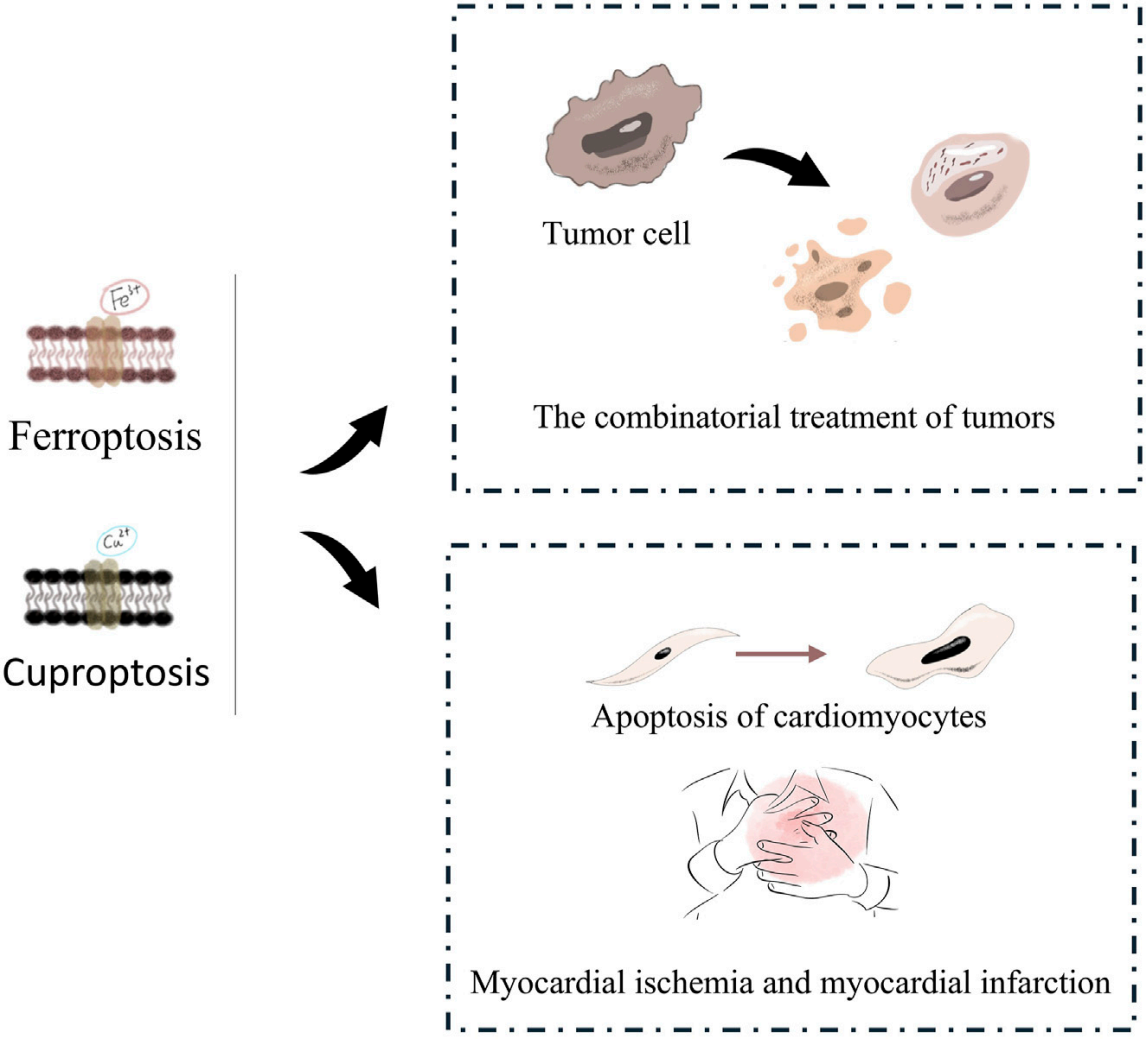
Cardiovascular risk of tumors initiated Ferroptosis or Cuproptosis treatment. On one hand, ferroptosis and cuproptosis can be synergistically used in combined tumor therapy to induce the death of tumor cells. On the other hand, they are also involved in the apoptosis of cardiomyocytes and are related to cardiovascular diseases such as myocardial ischemia and myocardial infarction.

**FIGURE 6 F6:**
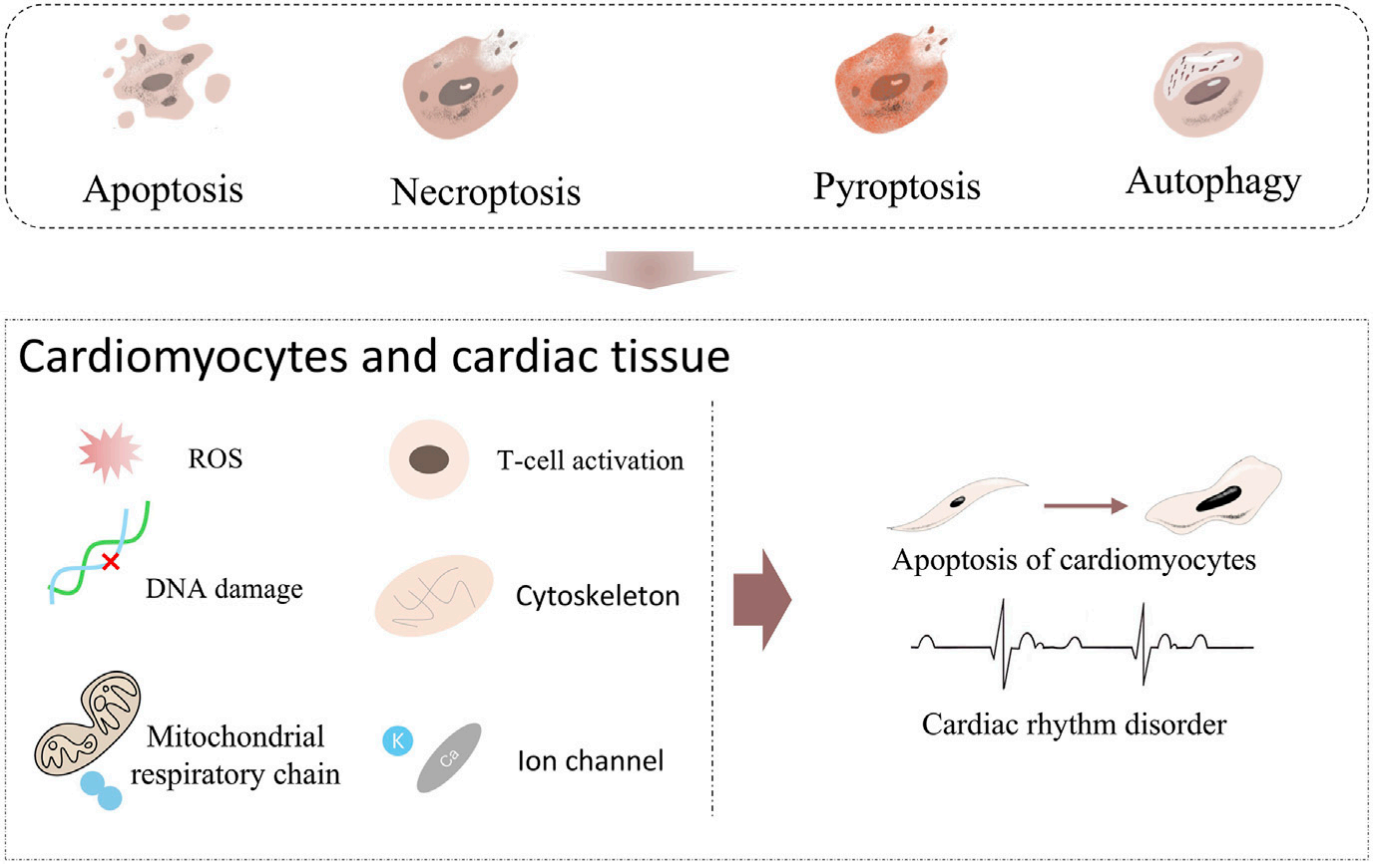
Known or potential cardiovascular risks of Apoptosis, Necroptosis, Pyroptosis and Autophagy. Apoptosis, Necroptosis, Pyroptosis, and Autophagy. When these forms of cell death act on cardiomyocytes and cardiac tissue, they trigger a series of injury inducers. For example, ROS induces oxidative stress, DNA damage destroys genetic material, T-cell activation initiates immune attack, cytoskeleton abnormality affects cell structure, mitochondrial respiratory chain malfunction interferes with energy metabolism, and ion channel abnormality disrupts electrophysiological activity. When these inducers act continuously, they will drive cardiomyocytes towards Apoptosis and further lead to Cardiac rhythm disorder.

Chemotherapeutic drugs are one of the most widely used and longest-established types of tumor treatment drugs (Zhou et al., 2023). Numerous existing studies have shown that anthracyclines (Hulst et al., 2022), taxanes (Sun et al., 2022), and alkylating agents (Kreher et al., 2023) all exhibit significant cardiotoxicity (Figures 5,6 [Fig F6]). Anthracyclines represented by DOX, or epirubicin and daunorubicin, induce DNA damage by intercalating into DNA and inhibiting topoisomerase II, thereby activating cell apoptosis pathways (van der Voort et al., 2021). While DOX acts on cancer cells, it also binds to cardiac myocyte-specific topoisomerase II, leading to double-strand breaks in myocardial nuclear DNA. The DNA damage is difficult to repair, triggering the p53-dependent apoptosis pathway (Büyükçelik et al., 2005; Lin et al., 2022). Additionally, after entering cardiac myocytes, DOX intercalates into the mitochondrial membrane lipid bilayer and disrupts the mitochondrial electron transport chain, leading to electron leakage and subsequent production and release of reactive oxygen species (ROS) (Kim and Choi, 2021; Xie et al., 2022). ROS induces the expression of Bcl-2 associated X protein (Bax)/Bcl-2 homologous antagonist/killer protein (Bak), leading to increased mitochondrial membrane permeability. Subsequently, cytochrome C is released to activate Caspase-9, which in turn activates Caspase-3 to execute the apoptotic process of cardiac myocytes (Chang et al., 2023; Shen et al., 2021).

#### Anthracyclines

**FIGURE 7:**
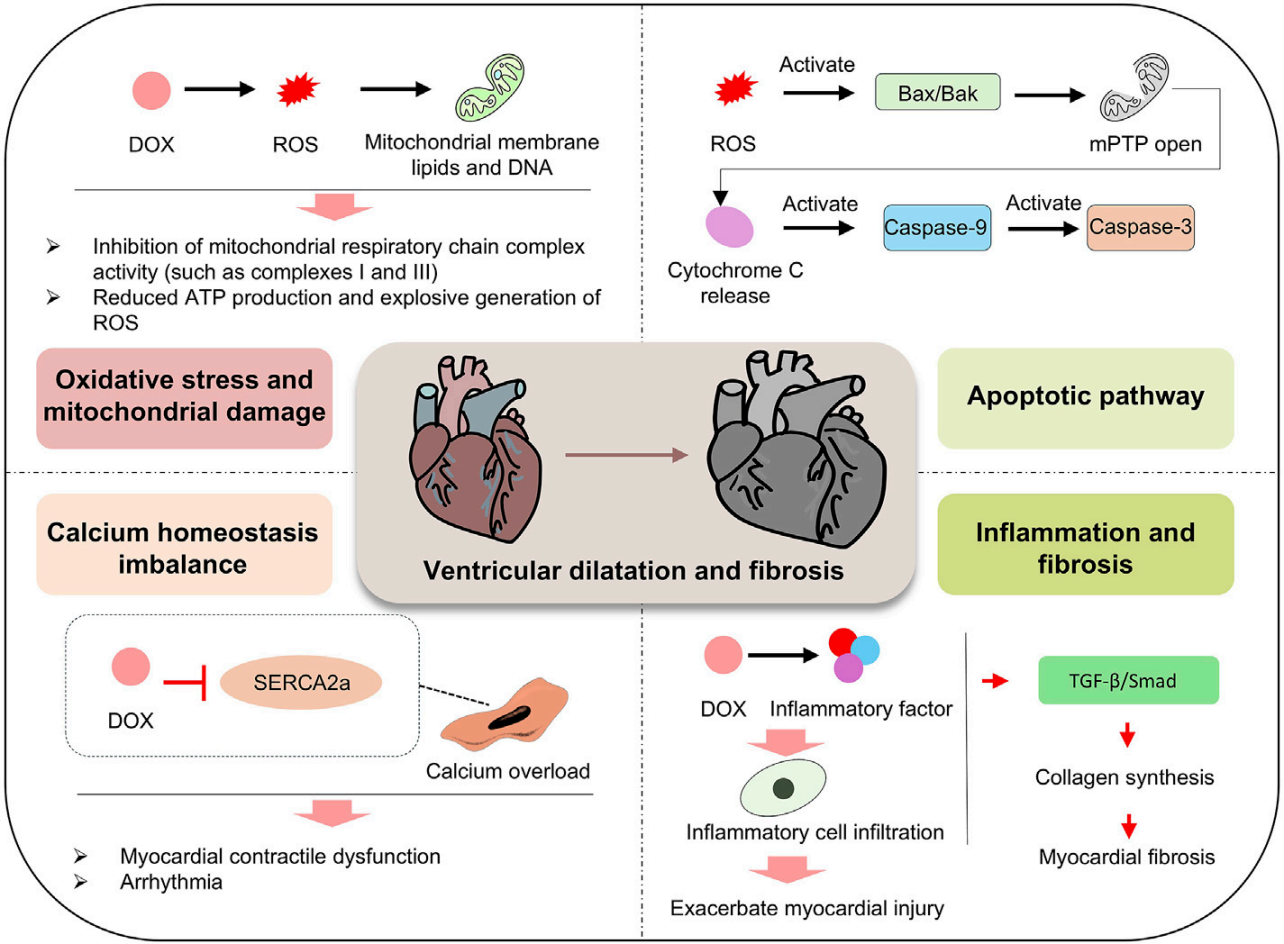
Mechanism of DOX effects on the heart. DOX promotes the generation of ROS, which damages mitochondrial membrane lipids and DNA, inhibits the activity of mitochondrial respiratory chain complexes (such as Complex I and III), reduces ATP production, and trigger a burst of ROS, leading to oxidative stress and mitochondrial damage. Concurrently, DOX inhibits SERCA2a, disrupts calcium homeostasis, causes calcium overload, and induces myocardial contractile dysfunction and arrhythmias. In the apoptotic pathway, ROS activate Bax/Bak, leading to the opening of the mitochondrial permeability transition pore (mPTP), the release of cytochrome c, and the sequential activation of Caspase-9 and Caspase-3. In inflammatory and fibrotic pathways, DOX induces the release of inflammatory factors, infiltration of inflammatory cells, and promotes collagen synthesis via the TGF-β/Smad pathway, exacerbating myocardial fibrosis. These multiple, potentially synergistic mechanisms contribute to ventricular dilation and fibrosis, driving the progression of myocardial injury.

Anthracyclines have been widely used in clinical cancer treatment. According to the timing of cardiac toxicity appearance, they can be classified into acute toxicity, subacute toxicity, and chronic toxicity (Cai et al., 2019; Curigliano et al., 2010). Acute toxicity occurs within hours to days after drug administration, caused by transient myocardial cell dysfunction due to a massive burst of ROS in the short term (Cai et al., 2019). Subacute myocardial toxicity occurs within weeks to months after drug administration. During this phase, persistent accumulation of ROS leads to severe DNA damage in myocardial cells, triggering myocardial cell apoptosis and partial myocardial necrosis (Dempke et al., 2023). Chronic toxicity occurs years after drug administration or even persists lifelong. In contrast to the former two, chronic toxicity involves severe myocardial cell loss and long-term cardiac fibrosis, leading to irreversible cardiac dysfunction such as dilated cardiomyopathy and New York Heart Association (NYHA) class III-IV HF (Figure 7) (Xie et al., 2024). In clinical use of anthracyclines, cardiac function changes must be monitored via echocardiography, B-type natriuretic peptide (BNP) levels, troponin levels, and electrocardiography. Interventions should be initiated when significant changes in indicators of HF risk occur, including angiotensin-converting enzyme inhibitors (ACEIs)/angiotensin receptor blockers (ARBs), β-blockers, spironolactone, and sodium-glucose cotransporter 2 (SGLT-2) inhibitors (Porter et al., 2022).

To reduce the cardiac damage caused by anthracycline chemotherapy, several clinical application protocols and preclinical studies have made multi-faceted efforts. On the one hand, it is about how to reduce the cardiotoxicity of anthracyclines under the premise of ensuring chemotherapeutic efficacy, and on the other hand, how to enhance the heart’s ability to cope with anthracyclines (Cejas et al., 2024). Clinical practice often involves replacing short-term high-dose administration of DOX with continuous infusion or fractional dosing to reduce its cardiotoxicity (Pollack et al., 2020). Preclinical studies aim to alter the *in vivo* distribution of drugs to reduce the cardiotoxicity of anthracyclines, including some newly developed drug delivery systems such as DOX liposomes and DOX nanoparticles (Ding et al., 2024; Yang et al., 2024). Some cardioprotective drugs are commonly used to protect the heart from anthracycline-induced damage, typically by influencing the redox metabolic processes of cardiac myocytes. Take Dexrazoxane as an example: it chelates iron ions from the Fe^3+^-anthracycline complex, inhibits hydroxyl radical formation, and directly scavenges superoxide anions (Chow et al., 2023; Huang et al., 2024). Reduced GSH is another commonly used reducing agent. In DOX-induced cardiotoxicity, GSH reduces DOX cardiac toxicity by enhancing superoxide dismutase (SOD) activity and decreasing the expression of Bax/Bcl-2 apoptotic signaling (Huang et al., 2024).

#### Taxanes

Taxanes, like anthracyclines, also lead to increased of ROS in cardiac myocytes, primarily superoxide anions (O_2_^−^) and hydrogen peroxide (H_2_O_2_). Generally, the cardiotoxicity of taxanes is much less severe than that of anthracyclines (Mosca et al., 2021; Xiao et al., 2021b). However, taxanes do affect the mitochondrial fusion-fission process (You et al., 2021). For example, docetaxel induces dynamin-related protein 1 (Drp1)-mediated abnormal mitochondrial fission, leading to collapse of the mitochondrial membrane potential (ΔΨm) and a 50% increase in cytochrome c release (Xiong et al., 2024). Taxanes also affect cellular structure by stabilizing microtubules and blocking mitosis to induce tumor cell apoptosis, while simultaneously interacting with cardiac tubulin to disrupt cytoskeletal integrity and trigger myocardial cell apoptosis (Das et al., 2021). All the above effects on cardiac myocytes can lead to myocardial cell loss and trigger cardiac dysfunction, manifested as reduced left ventricular ejection fraction and congestive HF (Lu et al., 2025).

Unlike anthracyclines, taxanes also affect the normal rhythm of the heart. Some studies have shown that paclitaxel can directly bind to the human ether-a-go-go related gene (hERG) channel (a potassium ion channel) in cardiac myocytes and prolong the action potential, leading to QT interval prolongation (Rathkopf et al., 2025). When paclitaxel acts on Ca^2+^-ATPase, it can cause intracellular Ca^2+^ overload in cardiac myocytes and lead to cardiac rhythm abnormalities, manifested as sinus bradycardia or supraventricular tachycardia (Wang et al., 2022). In addition, histamine release by taxanes can cause vasodilation and hypotension, leading to acute cardiovascular disorders or chronic cardiac injury (Tsao et al., 2022).

#### Alkylating agents

Alkylating agents are one of the earliest and most widely used anticancer drugs. Compared with anthracycline anticancer drugs, alkylating agents have a lower incidence of cardiotoxicity (Highley et al., 2022). Clinically, alkylating agents associated with cardiotoxicity mainly include Cyclophosphamide (CTX) (Fanouriakis et al., 2021), Ifosfamide (IFO) (Emami et al., 2024), and platinum compounds such as CDDP (Oaknin et al., 2024) and Carboplatin (CBP) (Baas et al., 2021). CTX exhibits a certain probability of causing hemorrhagic myocarditis, typically occurring with high-dose use (>100 mg/kg). CDDP can directly damage cardiomyocytes. Upon entering cardiomyocytes, it binds to DNA or proteins, disrupting normal cellular metabolism. Since CDDP affects ion channels such as calcium and potassium channels, its administration is often accompanied by alterations in cardiomyocyte action potentials and repolarization, potentially leading to arrhythmias (Panopoulos et al., 2023; Pudil et al., 2020). Given their early use and well-established clinical application, details will not be repeated here.

#### Targeted drugs

Targeted therapies, particularly HER2 inhibitors like trastuzumab and pertuzumab, exhibit notable cardiotoxic potential. These agents block HER2-mediated oncogenic signaling and enhance antibody-dependent cellular cytotoxicity but also disrupt HER2-dependent survival pathways in cardiomyocytes, impairing myocardial repair and promoting apoptosis (Kirkham et al., 2022).

For the known cardiotoxicity of HER2 inhibitors, clinical practice generally allows for the occurrence of cardiotoxicity with certain drugs. Take trastuzumab as an example: echocardiography and troponin level monitoring are typically used to assess cardiotoxicity risk and determine whether continued use of HER2-targeted inhibitors is appropriate. When left ventricular ejection fraction (LVEF) significantly decreases (50% < LVEF ≤ 90%) and cardiac troponin levels increase, interventions are initiated with ACEIs/ARBs and/or β-blockers, accompanied by increased frequency of echocardiographic monitoring (Porter et al., 2022).

Although BCL-2 inhibitors exhibit promising clinical activity in oncology, they pose cardiovascular risks by antagonizing the anti-apoptotic function of BCL-2 in cardiac myocytes, disrupting energy metabolism, and inducing ventricular remodeling. Additionally, BCL-2 inhibition alters cardiac electrophysiology by regulating potassium channels and delaying repolarization, thereby prolonging the QT interval—a known risk factor for life-threatening arrhythmias (Gao et al., 2023; Zhang et al., 2016).

#### Immune checkpoint inhibitors

ICIs, while revolutionizing cancer treatment through immune activation, carry unique cardiovascular hazards. T cell-mediated immune attack on cardiomyocytes and systemic cytokine storms—characterized by acute surges in IL-6, IFN-γ, and other pro-inflammatory cytokines—can trigger myocarditis, cardiomyocyte apoptosis, and circulatory collapse with high mortality (Sun et al., 2024).

During clinical use of ICIs, electrocardiograms, troponin levels, echocardiograms, and follow-up with symptom change recording are required. Any abnormal test results necessitate further evaluation. In the presence of significant cardiotoxic symptoms such as myocarditis, pericarditis, arrhythmias, or ventricular dysfunction, prompt intervention with corticosteroid drugs is indicated. For cases refractory to initial steroids, consideration should be given to adding mycophenolate mofetil, infliximab, or antithymocyte globulin (Alexandre et al., 2020).

#### Other related channels

In addition, some drugs that are still in the research phase or may pose potential cardiovascular risks through intrapathway analysis also warrant attention. MLKL is a key executioner protein in the necroptosis pathway. However, other studies suggest that excessive MLKL activation may trigger oxidative stress in cardiomyocytes, resulting in necrotic cell death. This also implies that inducing tumor cell necroptosis carries potential cardiotoxic risks (Gao et al., 2023; Zhang et al., 2016). Pyroptosis exerts anti-tumor effects through inflammasome activation, eliciting robust immune responses. However, in cardiovascular research, excessive NLRP3 activation can exacerbate cardiac inflammation, increasing the release of IL-1β and IL-18, inducing cardiomyocyte apoptosis, and contributing to myocardial hypertrophy and fibrosis. Existing evidence suggests that limiting NLRP3 activity facilitates recovery in damaged cardiac tissue. Therefore, maintaining balanced pyroptosis activation is beneficial for cancer therapy, particularly in hematologic malignancies. Nonetheless, further research is needed to identify the optimal equilibrium for pyroptosis induction to avoid associated cardiovascular toxicity. CQ and HCQ directly affect the electrophysiological properties of cardiomyocytes, significantly influencing heart rhythm and causing QT interval prolongation—a risk exacerbated when combined with antiarrhythmic drugs (Kim et al., 2020; Sutanto and Heijman, 2020). This stems from their direct impact on potassium and calcium ion channels, prolonging myocardial repolarization and increasing intracellular calcium concentration. Additionally, these drugs exert significant effects on cardiomyocyte structure and metabolism: studies show that CQ and HCQ interfere with myocardial β-oxidation, promote intracellular fatty acid accumulation, disrupt cardiac energy supply, and induce myocardial cell damage (Ballet et al., 2022; Sutanto and Heijman, 2020).

## Conclusion and prospect

PCD has emerged as a crucial strategy in anti-tumor drug development. PCD encompasses diverse modalities such as apoptosis, necroptosis, pyroptosis, autophagy, ferroptosis, and cuproptosis, each with distinct molecular regulatory networks and hallmarks. Apoptosis, a caspase-dependent process, is regulated by endogenous, exogenous, and ER stress-induced pathways. Necroptosis is caspase-independent and involves RIPK1, RIPK3, and MLKL. Pyroptosis is pro-inflammatory and executed via canonical or non-canonical pathways. Autophagy is a lysosome-mediated process for intracellular component recycling, with macroautophagy, microautophagy, and CMA as subtypes. Ferroptosis is characterized by iron-dependent lipid peroxidation, and cuproptosis is copper-dependent.

Many anti-tumor agents, including chemotherapeutics, targeted drugs, and immune checkpoint inhibitors, exert effects through modulating PCD pathways. However, these agents are associated with significant cardiovascular risks. For example, anthracyclines cause cardiomyocyte damage through free radical generation; HER2 inhibitors disrupt cardiomyocyte survival pathways; ICIs can trigger myocarditis and cytokine storms. Necroptosis in tumors has a dual role, and its induction may carry cardiotoxic risks. Pyroptosis can both promote and suppress tumorigenesis, and excessive activation in the cardiovascular system can lead to cardiac inflammation. Autophagy-targeting drugs like CQ and HCQ have cardiovascular side effects such as QT interval prolongation and myocardial cell damage. Ferroptosis induction can enhance the efficacy of conventional cancer therapies, but the associated cardiovascular risks need to be further explored.

Future research is needed to comprehensively understand the complex interplay between different PCD pathways and cardiovascular biology. This will facilitate the development of more effective and safer cancer therapies. Strategies could include designing drugs that specifically target PCD pathways in tumor cells while minimizing cardiovascular toxicity. Additionally, personalized medicine approaches may be developed, considering individual patient’s genetic profiles and cardiovascular status to optimize treatment regimens. Moreover, exploring novel combinations of therapies that synergistically induce PCD in tumors while protecting the cardiovascular system holds great promise for improving cancer treatment outcomes.
